# Effect of Qingre Lishi decoction on anthropometric and metabolic risk indices in obese patients with newly diagnosed T2DM: a real-world observational study

**DOI:** 10.3389/fendo.2026.1721267

**Published:** 2026-01-21

**Authors:** Bingchen Wei, Tianshu Gao, Qingyang Liu

**Affiliations:** 1 The First Clinical College, Liaoning University of Traditional Chinese Medicine, Shenyang, Liaoning, China; 2 Endocrinology Department, the First Affiliated Hospital of Liaoning University of Traditional Chinese Medicine, Shenyang, Liaoning, China

**Keywords:** anthropometric indices, clinical research, metabolism, obesity, therapeutic efficacy observation, Traditional Chinese Medicine, treatment with Chinese herbal decoctions, type 2 diabetes mellitus

## Abstract

**Objective:**

We have found the Qingre Lishi decoction can effectively reduce blood glucose and mitigates glycemic fluctuations in patients with newly diagnosed type 2 diabetes mellitus (T2DM). However, its influence on body fat distribution and associated metabolic parameters remains underexplored. This study aims to provide more comprehensive and integrated evidence regarding the clinical efficacy of the Qingre Lishi decoction.

**Methods:**

We involved 70 overweight and obese patients with newly diagnosed T2DM between December 2021 and November 2022. Patients were allocated to the observation group (*n* = 35), who received the Qingre Lishi decoction add lifestyle intervention, or the control group (*n* = 35), who received lifestyle intervention only. Comparative analyses were included waist circumference (WC), body mass index (BMI), waist-to-height ratio (WHtR), a body shape index (ABSI), body roundness index (BRI), triglycerides (TG), low-density lipoprotein cholesterol (LDL-C), lipid accumulation product (LAP), the triglyceride-glucose (TyG) indices, and related indices (TyG-WC, TyG-BMI, TyG-WHtR).

**Results:**

Regarding anthropometric indices, the observation group demonstrated significant reductions in WC, BMI, and WHtR, with the decreases in WC and WHtR being significantly greater than those in the control group (*P* < 0.05). The novel indices, ABSI and BRI, also showed a significant downward trend in the observation group compared to the controls. In terms of body fat percentage, the observation group exhibited a significantly greater reduction than the control group (*P* < 0.01). Concerning lipid profiles, TG and LDL-C levels decreased in the observation group, with a significantly larger reduction in LDL-C compared to the control group (*P* < 0.05). Furthermore, the metabolic risk indices TyG, TyG-WC, TyG-BMI, and TyG-WHtR all decreased significantly in the observation group, and these reductions were markedly superior to those in the control group (*P* < 0.01).

**Conclusion:**

The therapeutic efficacy of the Qingre Lishi decoction in overweight and obese patients with newly diagnosed T2DM is multifaceted, demonstrated by its ability to enhance abdominal fat distribution, lower TG and LDL-C levels, and improve metabolic risk indices (TyG, TyG-WC, TyG-BMI, and TyG-WHtR) for a more integrated patient outcome.

## 1 Introduction

In recent years, the global prevalence of diabetes mellitus (DM) has continued to rise, with a corresponding increase in the number of patients in China concomitant overweight or obesity (1, 2). According to a cross-sectional survey, more than 60% of Chinese adults with DM were either overweight or obese, with 42.4% classified as overweight [24 kg/m²≤body mass index (BMI)<28 kg/m²] and 25.3% as obese (BMI≥28 kg/m²) (3). Currently, Type 2 diabetes (T2DM) is the prevailing form of diabetes in China, where overweight and obesity are established primary risk factors (4). A study of Chinese adults with T2DM revealed that 39.7% present with abdominal obesity [visceral fat area (VFA)≥100 cm²], a figure that rises to 49.8% among overweight patients and 83.1% among those with obesity (5). Therefore, for T2DM patients, clinical management should not only mind glycemic control, but also concern body composition and related metabolic disorders.

Nowadays, more and more clinical guidelines, including the American Diabetes Association (ADA) (2025 version), the American Association of Clinical Endocrinology (AACE) (2023 version), and various Chinese expert consensus or statements, emphasize the necessity of moving beyond a singular focus on glycemic reduction. They pointed out that clinicians should also pay attention to the improvement of blood pressure, lipids, weight and other indicators, so as to provide patients with a more comprehensive and integrated individualised treatment plan (4, 6–9). Traditional Chinese Medicine (TCM), on the other hand, has a long history of treating DM due to its unique diagnostic and therapeutic modalities, and is expected to bring a new dawn to patients.

T2DM belongs to the category of Wasting-thirst (Xiao Ke) in TCM, and its etiology, pathogenesis and clinical symptoms can be traced back to the *Huangdi Neijing*. This book described the main symptoms of T2DM, including thirst and sweetness in the mouth. It further attributed the etiology of the disease primarily to the pathological patterns of Phlegm-damp and Damp-heat. In addition, TCM has evolved in the long course of clinical practice. It enables Chinese medicine not only to improve glycemic control but also to alleviate clinical symptoms, optimize body fat distribution, and synergistically reduce key metabolic markers such as blood pressure, lipid levels, and uric acid (10–12). Previous studies from our group have established through preliminary studies that newly diagnosed T2DM patients predominantly present with the type of Damp-heat trapped spleen, and they are often overweight or obese. We have further demonstrated that this patient cohort exhibits severe oxidative stress-induced damage and markedly elevated activation of the RASS. Furthermore, our team has discovered that Chinese herb can effectively improve glycemic control, alleviate blood glucose fluctuations, and significantly enhance patients’ adherence and self-confidence in controlling blood glucose (13–16). Notably, our clinical observations have also revealed that in addition to regulating blood glucose, Chinese herb decoctions exert a beneficial effect on the patients’ body fat distribution and lipid profiles.

Based on the above background, the present article analyses the real-world data collected in the previous period, aiming to explore the efficacy of the Qingre Lishi decoction on body composition management and lipid metabolism of obese patients with newly diagnosed T2DM. Compared with the commonly used clinical indicators such as waist circumference (WC) and BMI, this analysis introduced other novel anthropometric indices and metabolic risk indices, such as a body shape index (ABSI), body roundness index (BRI), lipid accumulation product (LAP), triglyceride-glucose (TyG) and related indices, which provided a more comprehensive perspective for evaluating the efficacy of the Qingre Lishi decoction (17).

## 2 Methods

### 2.1 Study participants

This was a retrospective real-world study. Data for all patients were extracted from paper-based medical records and outpatient electronic health systems by the team of professionally trained physicians to compile the study’s dataset. We used consecutive sampling to ensure that all eligible patients meeting the inclusion and exclusion criteria between December 2021 and November 2022 in the outpatient clinic were included in the study. To be included, all patients had to meet the diagnostic criteria for T2DM and satisfy the following conditions: (1) age between 18 and 65 years, regardless of gender; (2) overweight and obese, BMI≥24kg/m² [defined by Chinese criteria (18)]; (3) the disease duration of ≤ 12 months; (4) no COVID-19 infections in the last 6 months, 48 hours negative for novel coronavirus-N gene test and negative for novel coronavirus-ORF1ab gene test.

We also excluded patients with any of the following exclusion criteria: (1) failure to meet the new diagnosis of T2DM; (2) women who are pregnant or breastfeeding; (3) those with severe heart, lung, brain, liver and kidney diseases; (4) combination of any diabetic comorbidities and complications of diabetes mellitus; (5) allergy or intolerance to therapeutic drugs; (6) severe mental disorders, functional neurologic disorders and inability to communicate properly; (7) other diseases that may have an effect on glucose metabolism; (8) experience of a critical illness or other stressful situation within the last month; (9) participation in other studies within the last 3 months; (10) COVID-19 infection in the last 6 months, or 48 hours positive for novel coronavirus-N gene and positive for novel coronavirus-ORF1ab gene.

The final study comprised 70 overweight and obese patients with newly diagnosed T2DM in the Department of Endocrinology of the Affiliated Hospital of Liaoning University of Traditional Chinese Medicine between December 2021 and November 2022. This trial was approved by Ethics Committee of the First Affiliated Hospital of Liaoning University of Traditional Chinese Medicine [Y2023109CS(KT)-109-01, location: Room 236, Research Building, Liaoning Traditional Chinese Medicine Rehabilitation Centre, No.72 Chongshan East Road, Huanggu District, Shenyang] and was registered in the International Traditional Medicine Clinical Trial Registry (ITMCTR2024000006, registration date: 15/01/2024).

### 2.2 Study design

According to the patients’ voluntary choices during the clinical practice, they were allocated into two groups: those who chose to receive the Qingre Lishi decoction in combination with standardized lifestyle modification were assigned to the observation group, while those who chose to receive lifestyle intervention alone and declined pharmacological treatment were assigned to the control group. The standardized lifestyle modification was defined as the provision of DM health education, training in continuous glucose monitoring (CGM) use, and instruction on a dedicated mobile application for all enrolled patients. CGM was required for all patients throughout the treatment period. Study team members provided each patient with T2DM dietary guidelines, recommending a calorie intake of 30 kcal/(kg·d) for three meals, with 50 per cent carbohydrates, 15 per cent protein and 35 per cent fat (19). Furthermore, patients were instructed to abstain from smoking and alcohol consumption. For exercise guidance, patients were advised to engage in 30 minutes of slow walking each morning.

The patients in the observation group were additionally given the treatment of the Qingre Lishi decoction. The main medicine composition: radix bupleuri (Chai Hu) 15g, rhizoma pinellinae praeparata (Fa Banxia) 15g, scutellaria baicalensis (Huang Qin) 15g, wine-treated rhubarb (Jiu Dahuang) 15g, sinocalamus affinis (Zhu Ru) 15g, fructus aurantii immaturus rhizome (Zhi Shi) 10g, anemarrhenae (Zhi Mu) 10g, raw gypsum (Sheng Shigao) 15g, coptis chinensis (Huang Lian) 15g, cassia twig (Gui Zhi) 10g, rhizoma zingiberis (Gan Jiang) 10g, dark plum (Wu Mei) 5g, schisandra chinensis (Wu Weizi) 5g. Preparation and administration: The aforementioned Chinese herbal formula was decocted in water daily to yield a 300 mL preparation. Patients were instructed to take 100 mL of the warm decoction three times a day, with meals. At the same time, the herbal prescription was individualized and adjusted throughout the treatment based on syndrome differentiation of the patient’s clinical symptoms.

All patients accepted baseline assessments to confirm their diagnosis of T2DM. These included the measurement of height, body weight, WC, an oral glucose tolerance test (OGTT), and the collection of fasting venous blood samples. Follow-up evaluations were conducted at the 0d, 14d, and 28d to monitor changes in body weight, WC, CGM monitoring indicators, lipid profiles, as well as safety indicators. Any adverse events were systematically recorded throughout the study period.

### 2.3 Outcome measures

#### 2.3.1 Anthropometric indices

The anthropometric indices involved in this analysis include basic anthropometric indices and novel anthropometric indices. The basic anthropometric indices included height (cm), body weight (kg), WC (cm), BMI (kg/m2), and waist-to-height ratio (WHtR). The anthropometric measurements were obtained from all patients before the treatment. Height and body weight were measured using an integrated digital stadiometer and scale, with readings respectively recorded to the nearest 0.1 cm and 0.1 kg. WC was measured according to a standardized protocol [World Health Organization (WHO) recommended method]. With the patient in a standing position, the measurement was taken at the midpoint between the lower margin of the twelfth rib and the anterior superior iliac spine. A flexible, non-stretchable measuring tape was placed horizontally around the abdomen at this level, ensuring it was snug against the skin without causing compression. The WC value was recorded at the end of a normal expiration to the nearest 0.1 cm. BMI was calculated according to the formula:


$$BMI\;(kg/m^{2})\;=\;\frac{weight\;(kg)}{{\left[height\;(m)\right]}^{2}}$$

WHtR was calculated according to the formula:


$$WHtR\;=\;\frac{WC\;(cm)}{height\;(cm)}$$

Recognizing the inherent limitations of anthropometric indices such as BMI and WC, this study incorporates novel indices to provide a more comprehensive assessment of the therapeutic effects of the Qingre Lishi decoction (2, 20–22). One such indicator is ABSI, which was first proposed by Krakauer et al. in 2012 (22). ABSI standardizes WC relative to both BMI and height; consequently, a higher ABSI value signifies a larger WC for a given body mass and stature, reflecting a visceral obesity phenotype. Given our focus on a Chinese population, we utilized the specific ABSI formulas developed for Chinese adults by Wang Hongli et al. (23). The formula used for calculation was as follows (23):


$$ABSI\;(kg^{-0.731}m^{1.843})\;=\;\frac{WC\;(m)}{{\left[BMI(kg/m^{2})\right]}^{0.731}\;\times height\;{\left(m\right)}^{0.619}}$$

Additionally, BRI was developed by Thomas et al. in 2013, and it was utilized to estimate visceral adipose tissue. A higher BRI value indicates greater visceral fat accumulation (24). The BRI was calculated using the following formula (25):


$$BRI=364.2-365.5\times \sqrt{1-\left\{\frac{{\left[WC\;\left(m\right)/2\pi \right]}^{2}}{{\left[0.5\times height\;\left(m\right)\right]}^{2}}\right\}}$$

#### 2.3.2 Glucose and lipid indicators and safety indices

The glucose and lipid indices were measured at the 0d. They were taken from venous blood samples, drawn from the cubital vein after patients completed an OGTT test, including fasting blood glucose (FPG, mmol/L), 2-hour postprandial blood glucose (2hPG, mmol/L), glycated hemoglobin (HbA1c, %), fasting C-peptide (FCP, ng/mL), triglycerides (TG, mmol/L), and low density lipoprotein cholesterol (LDL-C, mmol/L). Follow-up assessments at 14d and 28d included FPG, 2hPG, and estimated HbA1c (eHbA1c, %) that provided by CGM. TG and LDL-C were measured at 28d. Safety indicators included alanine aminotransferase (ALT, U/L), aspartate aminotransferase (AST, U/L), γ-glutamyl transpeptidase (GGT, U/L), serum creatinine (Scr, μmol/L), urea nitrogen (UREA, mmol/L) at 0d and 28d.

FPG, TG, LDL-C, ALT, AST, GGT, Scr, and UREA were measured by automated biochemical analyzer (Hitachi Model 7600-020; Tokyo, Japan). The institutional normal reference ranges for these indicators were as follows: FPG, 3.9 ~ 6.1 mmol/L; TG, 0.7 ~ 1.7 mmol/L; LDL-C, ≤ 3.62 mmol/L; ALT, 5 ~ 40 U/L; AST, 8 ~ 40 U/L; GGT, 11 ~ 50 U/L; Scr, 59 ~ 104 µmol/L; and UREA, 2.9 ~ 8.2 mmol/L. Levels of HbA1c and FCP were measured by electrochemiluminescence immunoassay (Cobas e601; Roche, Germany). The institutional normal reference ranges for these indicators were as follows: HbA1c, 4.0% ~ 6.0%, FCP, 1.1 ~ 4.4 ng/mL.

Blood glucose levels were monitored using the GS1 Continuous Glucose Monitoring System (Shenzhen Silicon-Based Sensing Technology Co., Ltd., Shenzhen, China; NMPA Registration No. 20213070871). The system includes a sensor and the Silicon-based Dynamic mobile app (version 01.11.00.00).

#### 2.3.3 Metabolic risk indices

In recent years, a series of novel metabolic risk indices have gained significant attention. These indices offer considerable advantages and represent a promising approach for assessing metabolic dysfunction, insulin resistance (IR), DM, and arteriosclerotic cardiovascular disease (ASCVD) risks (26–28).

Therefore, to comprehensively investigate the impact of the Qingre Lishi decoction on the metabolic state of overweight or obese patients with newly diagnosed T2DM, we calculated LAP, TyG-related indices using baseline data of WC and TG. This was aimed to thoroughly evaluate the therapeutic efficacy of the Qingre Lishi decoction. The LAP index, first proposed by Kahn et al. in 2005, combines WC and TG to reflect lipid accumulation and assess diabetes risk (29). It was calculated with the following sex-specific formulas (25):

For males:


LAP = [WC (cm) - 65] × TG (mmol/L)

For females:


LAP = [WC (cm) - 58] × TG (mmol/L)

TyG-related indices (TyG, TyG-WC, TyG-BMI, and TyG-WHtR) have been shown to be significant predictors for IR, DM, and ASCVD (30–32). They were calculated with the following formulas:


$$TyG\;=\ln\left[\frac{TG\;(mmol/L)\;\times \;FPG\;(mmol/L)}{2}\right]$$

TyG-WC = TyG × WC (cm)

$$TyG-BMI\;=\;TyG\;\times \;BMI(kg/m^{2})$$

TyG-WHtR = TyG × WHtR

In this study, all metrics were rechecked by “Medical Calculators, Scores, and Scales” function available on the MedSci online platform (https://m.medsci.cn/scale/index.do). We combined the basic indices with the novel indices to investigate the effects of the decoction on weight management and metabolic indicators, aiming to evaluate the clinical efficacy of the Qingre Lishi decoction in a more comprehensive and systematic way.

### 2.4 Statistical analysis

The study used SPSS 25.0 statistical software to process the data. Quantitative data were tested for normality using the Shapiro-Wilk test. Normally distributed data was expressed by ( $\bar{x}$ ± s), and non-normally distributed data was expressed by [M(Ql,Qu)]. For categorical data, we performed the Chi-square test. For between-group comparisons, independent samples *t* test was used for normally distributed quantitative data. Otherwise, Wilcoxon rank-sum test was applied. For within-group comparisons, paired *t* tests was used for normally distributed quantitative data. Otherwise, Wilcoxon paired rank-sum test was applied. Finally, *P* < 0.05 was considered statistically significant, and *P* < 0.01 was considered highly statistically significant.

In addition, to comprehensively evaluate the intervention effects, this study incorporated effect sizes in the within-group comparisons. Effect size quantifies the actual magnitude of the differences between groups, which is used to analyze the underlying trends in cases where statistical significance is not met, thus providing a more clinically meaningful interpretation of the results. For normally distributed data, the effect size was calculated using Cohen’s *d*. According to Cohen’s criteria, the effect size was *d*≥0.2 indicating a small effect; *d*≥0.5 indicating a medium effect, and *d*≥0.8 indicating a large effect. For non-normally distributed data, the effect size was calculated using Cliff’s Δ. The criteria for determining the effect size was Δ<0.147 indicating no effect, Δ≥0.147 indicating a small effect, Δ≥0.330 indicating a medium effect, and Δ≥0.474 indicating a large effect.

## 3 Results

Ultimately, 53 patients were included in the final analysis, comprising 31 patients in the observation group and 22 in the control group. Throughout the study period, 4 patients from the observation group and 13 from the control group were lost to follow-up. The reasons for withdrawal and the patient enrollment process are detailed in **Supplementary Figure S1**. To ensure group comparability, baseline characteristics of both groups were statistically analyzed prior to the intervention. The results indicated that there were no statistically significant differences in any baseline characteristics between the two groups (**Supplementary Tables S1**, **S2**).

### 3.1 Changes in anthropometric indices

#### 3.1.1 Changes in basic anthropometric indices

Regarding basic anthropometric indices, distinct patterns of change were observed between the two groups. Patients in the observation group exhibited a more pronounced decreasing trend in BMI, WC, and WHtR compared to the control group. At the 28d, WC and WHtR in the observation group were significantly lower than those in the control group (*P* < 0.05). This suggests that the Qingre Lishi decoction may possess a notable advantage in reducing WC and improving WHtR. Within-group comparisons revealed that in the observation group, WC, BMI, and WHtR were significantly reduced from baseline by the 14d (*P* < 0.01). These indices continued to decline, showing further significant reductions at the 28d compared to both the 0d and 14d (*P* < 0.01). In contrast, while the indices in the control group also showed significant reductions at the 14d and 28d compared to baseline (*P* < 0.01), the change between the 28d and 14d, despite a slight downward trend, was not statistically significant (*P*>0.05) (**Table 1**).

**Table 1:** Comparison of changes in anthropometric indices before and after observation between the two groups ( $\bar{x}$ ± s). .

Parameters	Observation time point (d)	Observation group	Control group	*t*	*P* value	Cohen’s *d*
WC (cm)	0	96.28 ± 9.42	96.03 ± 7.91	0.101	0.920	0.027
	14	94.16 ± 8.49*	92.70 ± 6.91*	0.666	0.509	0.191
	28	87.99 ± 6.47*^†^	91.67 ± 5.11*	-2.215	0.031^#^	0.495
BMI (kg/m^2^)	0	27.00 ± 2.34	27.40 ± 2.49	-0.604	0.549	0.172
	14	26.77 ± 2.08*	26.97 ± 2.36*	-0.322	0.749	0.087
	28	25.95 ± 1.71*^†^	26.61 ± 2.20*^†^	-1.225	0.226	0.340
WHtR	0	0.57 ± 0.04	0.57 ± 0.04	0.098	0.922	0.000
	14	0.55 ± 0.04*	0.55 ± 0.03*	0.877	0.384	0.120
	28	0.52 ± 0.03*^†^	0.54 ± 0.03*	-2.434	0.018^#^	0.604
ABSI (kg^-2/3^m^6/11^)	0	0.0726 ± 0.0076	0.0730 ± 0.0086	-0.182	0.857	0.047
	14	0.0711 ± 0.0065*	0.0699 ± 0.0084*	0.565	0.575	0.155
	28	0.0677 ± 0.0054*^†^	0.0694 ± 0.0065*	-1.010	0.317	0.271
BRI	0	4.94 ± 0.47	4.93 ± 0.60	0.060	0.953	0.017
	14	4.81 ± 0.50*	4.71 ± 0.50*	0.708	0.482	0.200
	28	4.42 ± 0.55*^†^	4.65 ± 0.50*	-1.559	0.125	0.421

^#^*P* < 0.05 compared the observation group with the control group; *P < 0.01 compared with the 0d of own group; ^†^*P* < 0.01 compared with the 14d of own group. WC, waist circumference; BMI, body mass index; WHtR, waist-to-height ratio; ABSI, a body shape index; BRI, body roundness index.

#### 3.1.2 Changes in novel anthropometric indices

For novel anthropometric indices, to more precisely capture subtle differences and avoid interpretations based on overly truncated data, ABSI values were retained to four decimal places. The results indicated that at the 28d, both ABSI and BRI in the observation group showed a downward trend compared to the control group. Although this within-group difference did not reach statistical significance, the effect sizes were 0.271 and 0.421, suggesting a potential influence of short-term intervention with the Qingre Lishi decoction on optimizing body composition (**Table 1**).

#### 3.1.3 Change value of anthropometric indices

**Figure 1:**
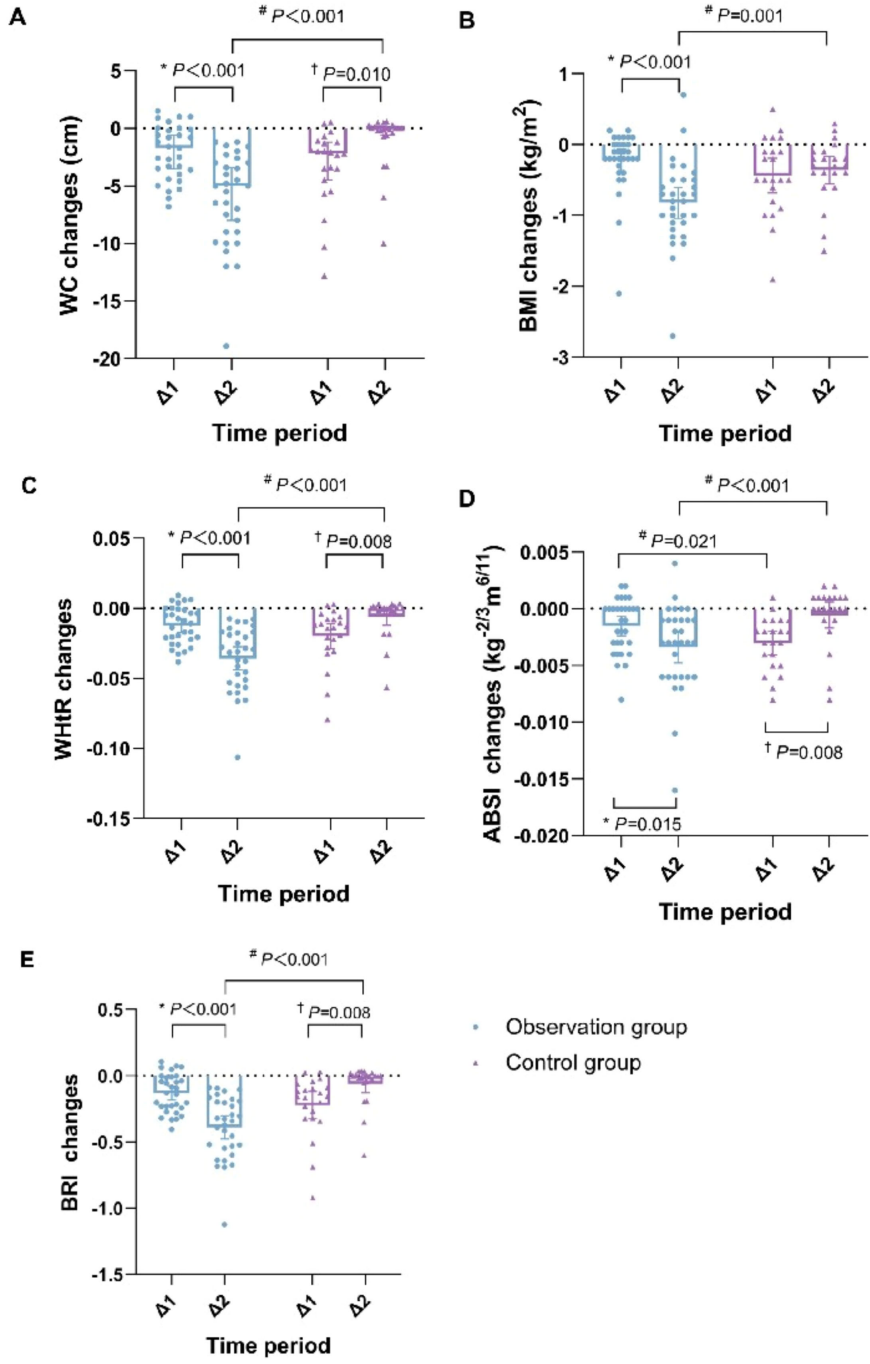
Comparison of changes in change value of anthropometric indices before and after observation between the two groups. **(A)** Change value of WC. **(B)** Change value of BMI. **(C)** Change value of WHtR. **(D)** Change value of ABSI. **(E)** Change value of BRI. ^#^*P* < 0.05 compared the observation group with the control group; *P < 0.05 compared with the Δ1 of the observation group; ^†^*P* < 0.05 compared with the Δ1 of the control group. Δ1: from 0d to 14d; Δ2: from 14d to 28d. WC, waist circumference; BMI, body mass index; WHtR, waist-to-height ratio; ABSI, a body shape index; BRI, body roundness index.

The change value of each indicator was compared between the two groups. We found that the differential changes in WC, BMI, WHtR, ABSI, and BRI in the observation group were statistically significant compared to the control group (*P* < 0.05), with the differential changes in ABSI being statistically significant in both time periods (Δ1: from 0d to 14d and Δ2: from 14d to 28d) (*P* < 0.05). Furthermore, an within-group analysis of the observation group showed that the improvements during the second time periods (Δ2: from 14d to 28d) were also statistically significant (*P* < 0.05), indicating sustained therapeutic effects (**Figure 1**).

### 3.2 Changes in body fat percentage

**Figure 2:**
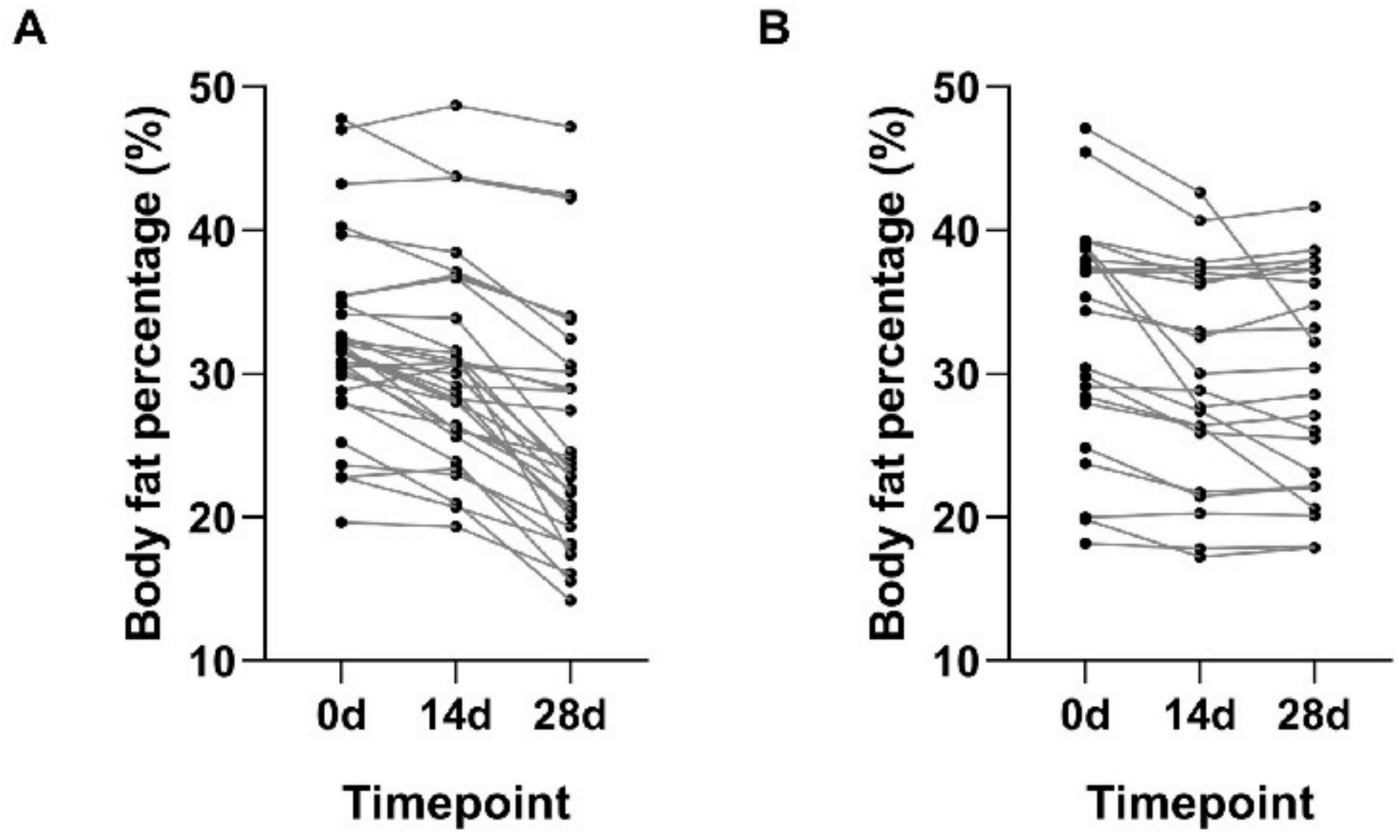
Changes in body fat percentage before and after observation between the two groups. **(A)** Changes of the observational group. **(B)** Changes of the control group.

There are various methods of calculating body fat percentage in humans, and we used waist circumference weight measurements to assess changes in body fat in patients at each observation time point. The results showed that the decrease in body fat percentage was more pronounced in the observation group (**Table 2**; **Figure 2**). Within this group, the mean body fat percentage at the 28d was significantly lower than the values recorded at both 0d and 14d (*P* < 0.01). In contrast, for the control group, a significant reduction in body fat percentage from baseline was observed at the 28d, but the change between the 14d and the 28d was not statistically significant (*P* < 0.01) (**Table 2**). These findings suggest that the Qingre Lishi decoction may have a preferential effect in accelerating the reduction of abdominal adiposity.

**Table 2:** Comparison of changes in body fat percentage before and after observation between the two groups [( $\bar{x}$ ± s) or M (Ql, Qu)].

Parameters	Observation time point (d)	Observation group	Control group	*t*	*P* value
Body fat percentage (%)	0	32.29 ± 6.50	32.76 ± 8.10	-0.234	0.816
	14	30.63 ± 7.03*	30.10 ± 7.54*	0.265	0.792
	28	26.03 ± 8.23*^†^	29.49 ± 7.59*	-1.556	0.126

*P < 0.01 compared with the 0d of own group; ^†^*P* < 0.01 compared with the 14d of own group.

### 3.3 Changes in lipid profile

To minimize patient burden, venous blood samples were collected under fasting conditions only at the 0d and 28d. These samples were used to assess TG, LDL-C, and LAP. The results indicated that the observation group showed an improving trend across all lipid markers, with all within-group changes from baseline being statistically significant (*P* < 0.01). More importantly, the reduction in LDL-C was significantly greater in the observation group compared to the control group (*P* < 0.05). This between-group difference was supported by a medium effect size (Cohen’s *d* = 0.620), suggesting a clinically meaningful therapeutic effect on LDL-C levels (**Table 3**).

**Table 3:** Comparison of changes in lipid profile before and after observation between the two groups [( $\bar{x}$ ± s) or M (Ql, Qu)].

Parameters	Observation time point (d)	Observation group	Control group	*t*/*Z*	*P* value	Cliff’s Δ/Cohen’s *d*
TG (mmol/L)	0	1.92 (1.25,3.25)	1.94 (1.07,2.68)	-0.370	0.711	-0.028
	28	1.46 (1.00,2.56)*	2.10 (1.54,2.34)	-1.643	0.100	-0.230
LDL-C (mmol/L)	0	2.83 ± 1.32	2.91 ± 0.99	-0.248	0.805	0.065
	28	2.07 ± 0.97*	2.61 ± 0.69	-2.272	0.027^#^	0.620
LAP	0	61.04 (44.47,101.93)	65.61 (39.60,89.83)	-0.144	0.885	-0.038
	28	36.50 (28.07,85.05)*	61.57 (42.18,74.77)	-1.516	0.129	-0.210

^#^*P* < 0.05 compared the observation group with the control group; *P < 0.05 compared with the 0d of own group. TG, Triglycerides; LDL-C, low-density lipoprotein cholesterol; LAP, lipid accumulation product.

### 3.4 Changes in TyG-related indices

**Figure 3:**
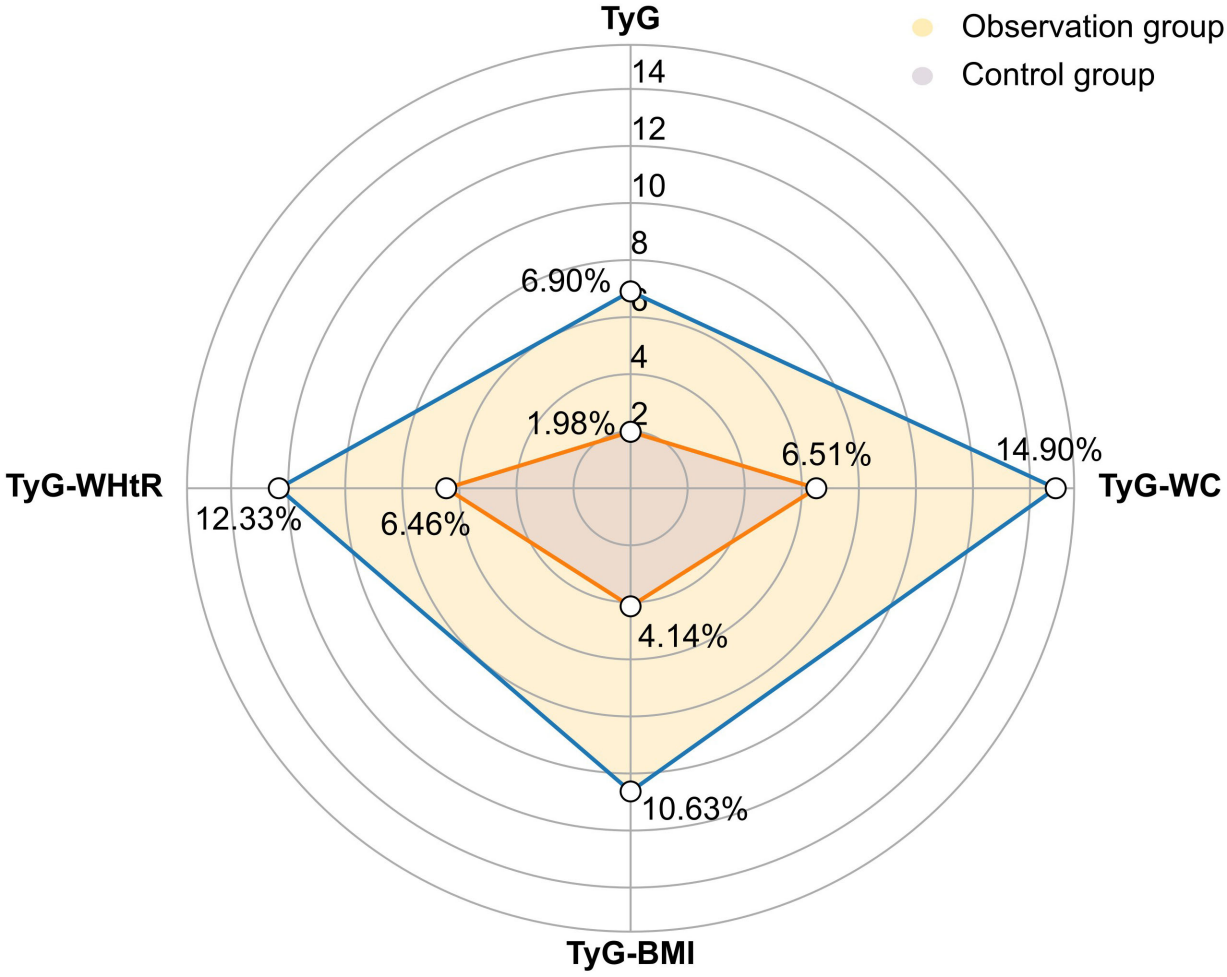
Percentage changes in TyG-related indices before and after observation between the two groups. TyG, Triglyceride-glucose; TyG-WC, TyG-Waist Circumference; TyG-BMI, TyG-Body Mass Index; TyG-WHtR, TyG-Waist-to-Height Ratio.

A comparative analysis of TyG-related indices was conducted. At the conclusion of the study, the observation group demonstrated a profound improvement across all measured indices. The values for TyG, TyG-WC, TyG-BMI and TyG-WHtR were all significantly lower compared to those in the control group (*P* < 0.01). To quantify the clinical relevance of these changes, within-group effect sizes were calculated. The reductions in TyG and TyG-BMI produced large effect sizes of 0.776 and 0.770, underscoring the substantial clinical efficacy of Qingre Lishi decoction in improving these indices. Notably, the effect sizes for TyG-WC and TyG-WHtR reaching 0.985 and 1.200, indicating a particularly significant efficacy in mitigating waist-related metabolic risks. Furthermore, within-group analysis of the observation group confirmed these positive outcomes, revealing that all four TyG-related indices at the 28d were significantly decreased from their baseline values (*P* < 0.01). Collectively, these findings strongly suggest that the Qingre Lishi decoction may significantly ameliorate IR and exert a beneficial regulatory influence on patients’ glucose and lipid metabolism (**Table 4**; **Figure 3**).

**Table 4:** Comparison of changes in TyG-related indices before and after observation between the two groups ( $\bar{x}$ ± s).

Parameters	Observation time point (d)	Observation group	Control group	*t*	*P* value	Cohen’s *d*
TyG	0	9.56 ± 0.67	9.58 ± 0.74	-0.112	0.912	0.027
	28	8.90 ± 0.60*	9.39 ± 0.44	-3.249	0.002^#^	0.776
TyG-WC	0	921.70 ± 122.10	920.80 ± 108.81	-0.028	0.978	0.008
	28	784.40 ± 88.28*	860.81 ± 65.29*	-3.443	0.001^#^	0.985
TyG-BMI	0	258.53 ± 33.40	260.69 ± 37.63	-0.220	0.827	0.060
	28	231.06 ± 24.60*	249.89 ± 24.45*	-2.753	0.008^#^	0.770
TyG-WHtR	0	5.27 ± 1.07	5.42 ± 0.55	-0.583	0.563	0.160
	28	4.62 ± 0.48*	5.07 ± 0.30*	-3.804	0.000^#^	1.200

^#^*P* < 0.01 compared the observation group with the control group; *P < 0.01 compared with the 0d of own group. TyG, Triglyceride-glucose; TyG-WC, TyG-Waist Circumference; TyG-BMI, TyG-Body Mass Index; TyG-WHtR, TyG-Waist-to-Height Ratio.

### 3.5 Safety indices

Throughout the study duration, ALT, AST, GGT, UREN and Scr of all patients in the observation group were within the normal range, and the differences between the follow-up points were not statistically significant (*P*>0.05). Regarding adverse events, the incidence of hypoglycemia was significantly lower in the observation group, where only two such events were recorded. The difference in incidence rate compared to the control group was statistically significant (*P* < 0.05) (**Supplementary Table S3**). All of the above results have been confirmed and published in previous analyses (16).

## 4 Discussion

Evidence indicates a rising trend of concurrent overweight or obesity among Chinese patients with T2DM. Compared to Caucasian counterparts, these patients exhibit a greater tendency for body fat to accumulate in the abdomen, leading to a higher prevalence of abdominal obesity (5, 33). Abdominal obesity, especially excessive accumulation of visceral fat, contributes to a substantially increased risk of IR and ASCVD in patients with T2DM (34, 35). However, BMI cannot accurately distinguish between fat and muscle, thus failing to accurately reflect the distribution of fat (36). Therefore, more and more expert consensus and studies have indicated that other indicators should be introduced to comprehensively assess the distribution of fat and evaluate the obesity status of patients, such as WC, WHtR, and ABSI (37–39).

**Figure 4:**
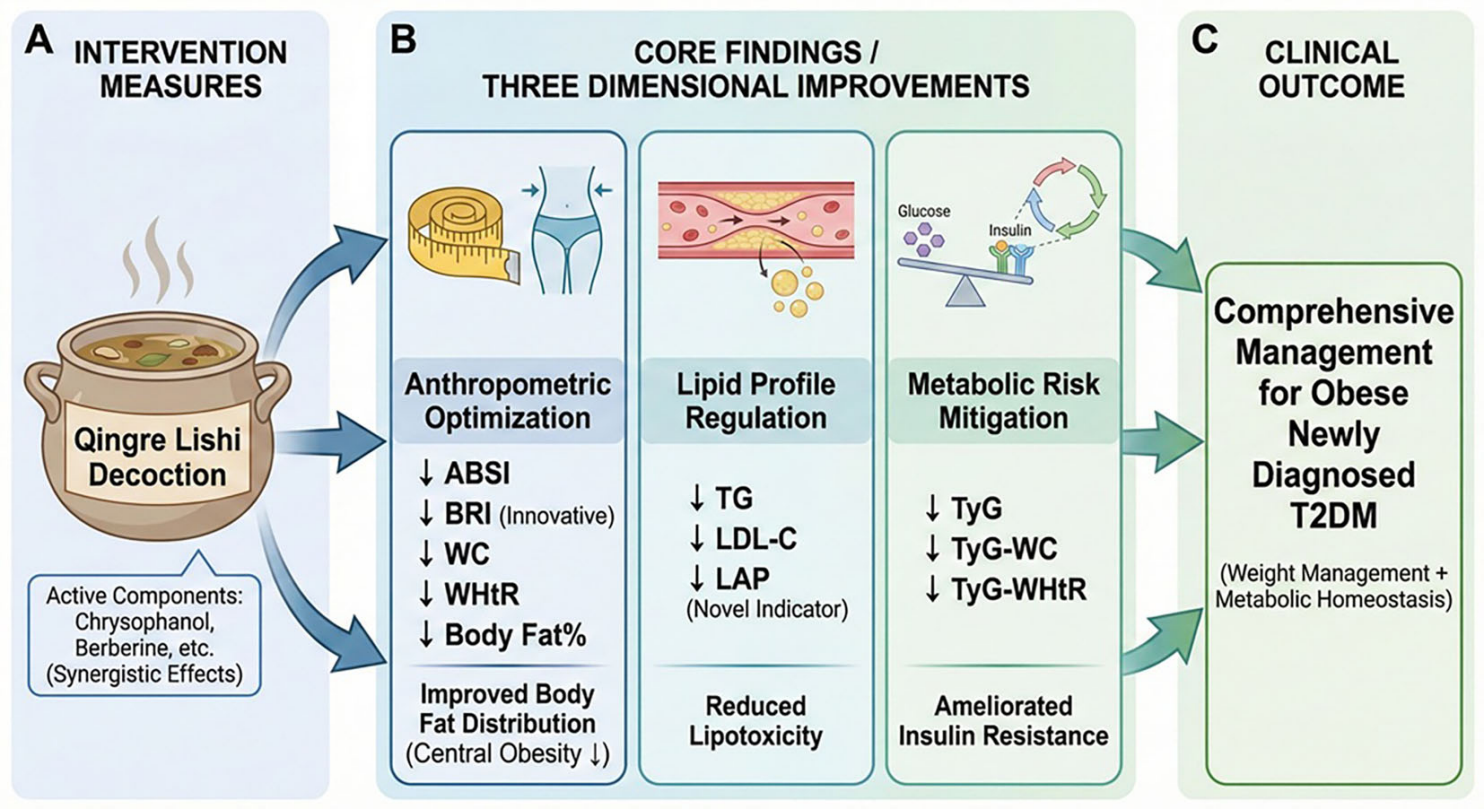
Schematic diagram illustrating the efficacy of the Qingre Lishi decoction on body fat distribution and metabolism in newly diagnosed obese patients with type 2 diabetes. **(A)** The intervention implemented in this study was Qingre Lishi Decoction. **(B)** This study revealed that Qingre Lishi Decoction improves three key aspects of body fat metabolism. **(C)** The clinical outcomes derived from this study. ABSI, a body shape index; BRI, body roundness index; WC, waist circumference; WHtR, waist-to-height ratio; TG, Triglycerides; LDL-C, low-density lipoprotein cholesterol; LAP, lipid accumulation product; TyG, Triglyceride-glucose; TyG-WC, TyG-Waist Circumference; TyGWHtR, TyG-Waist-to-Height Ratio.

Building upon our previous research, this study innovatively evaluates the effects of the Qingre Lishi decoction on body composition, lipid metabolism, and overall metabolic risk in obese patients with newly diagnosed T2DM. In addition to the basic anthropometric indices, we introduced WC and WHtR, which are more reflective of abdominal obesity, as well as the novel anthropometric indices ABSI and BRI (**Figure 4**). The results showed that compared with the control group, which received only lifestyle intervention, the observational group experienced a significant reduction in WC and WHtR at 28d, achieving a medium effect size. This suggests that the Qingre Lishi decoction may have a preferential effect on improving abdominal fat distribution. Furthermore, our analysis of the change values revealed that the rate of improvement in the observational group was significantly faster during the second time periods (Δ2: from 14d to 28d) than in the first time periods (Δ1: from 0d to 14d). In contrast, the improvements in the control group tended to stagnate. These findings indicate that the Qingre Lishi decoction have a sustained and cumulative effect, which may enable it to overcome the therapeutic plateau often observed in the later stages of lifestyle intervention alone.

Notably, although between-group comparisons of the endpoints for ABSI and BRI did not reach statistical significance, their effect sizes showed a small to moderate trend towards positive improvement. Analysis of the change values further indicated that the Qingre Lishi decoction was effective in improving body composition and reducing the risk of central obesity. We attribute these therapeutic outcomes to the pharmacological properties of the constituent herbs in the Qingre Lishi decoction. Modern pharmacological studies have validated that the therapeutic efficacy of traditional Chinese medicinal formulas, such as the Qingre Lishi decoction, originates from the harmonious synergy among their diverse active constituents. For example, modern pharmacological studies have shown that anthraquinone components such as chrysophanol and emodin in rhubarb (Da Huang) have the efficacy of regulating blood lipids, anti-inflammatory and antioxidant, and treating lipid metabolism disorders. Yuan Xinru et al. concluded that chrysophanol has the effects of lowering blood lipids and promoting fat metabolism. In addition, animal experiments have also shown that chrysophanol can reduce the levels of blood glucose and lipids in rats induced by a high-fat diet, increase the levels of fat degrading genes in rats, reduce the expression of adipokines, and decrease fat production (40, 41). Berberine, which is the highest content in coptis chinensis (Huang Lian) has been proved to have the effect of lowering blood glucose, regulating blood lipids, reducing obesity, and even improving lipid-induced insulin resistance (42–44). Furthermore, studies have also demonstrated that the combined application of rhubarb (Da Huang) and coptis chinensis (Huang Lian) can more effectively regulate glucose and lipid metabolism in rats, yielding superior clinical outcomes (45). Based on this, we infer that the efficacy of Qingre Lishi decoction also stems from the synergistic interactions between their herbal constituents. At present, our research team is employing methods such as network pharmacology, molecular docking, and machine learning to conduct animal experiments, aiming to predict and validate the synergistic effects among its active constituents.

Additionally, this study revealed that Qingre Lishi decoction beneficially lowered the lipid profile. This is particularly relevant as dyslipidaemia is a common comorbidity in patients with T2DM. Studies have shown that while LDL-C is a primary pathogenic driver of ASCVD, the risk of coronary artery disease (CAD) in the T2DM population is most significantly impacted by TG when LDL-C levels are also concomitantly elevated (46). Tsutomu Hirano further proposed the concept of “Atherogenic Duo” emphasizing the synergistic role of both LDL-C and TG in driving the development of atherosclerosis in patients with T2DM (47). Indeed, our preliminary data align with the existing literature, revealing that the therapeutic impact on LDL-C and TG is most significant in newly diagnosed T2DM patients (48, 49). For this reason, our study was designed to primarily measure these two indicators. We also recognized, however, that post-treatment changes in body fat distribution could alter the degree of visceral adiposity, thereby impacting factors such as IR. Therefore, to enable a more comprehensive assessment of the therapeutic efficacy of the Qingre Lishi decoction, we incorporated LAP and several TyG-related indices into our analysis.

A substantial body of recent research has established LAP as a sensitive and effective predictor for both diabetes mellitus and a variety of cardiovascular diseases (50, 51). Moreover, comparative meta-analyses have concluded that, when evaluated against other obesity indices, LAP is not only simple and accurate but also exhibits notable sex-specificity (52). The TyG-related indices, integrating lipid and glucose metrics, are validated proxies for insulin resistance and cardiovascular risk (53, 54). In summary, our analysis revealed that after 28 days, TyG, TyG-WC, TyG-BMI, and TyG-WHtR levels in the observation group were significantly lower than those in the control group, especially TyG-WC and TyG-WHtR levels. And this finding is also in keeping with our previous finding that the Qingre Lishi decoction markedly improved WC and WHtR, suggesting that it can change the distribution of abdominal fat.

We propose that the therapeutic effects of the Qingre Lishi decoction extend beyond a simple additive effect of glucose and lipid reduction. As a multi-component herbal formula, its action may fundamentally ameliorate intracellular glucolipotoxicity, a finding that aligns with animal studies on Jinlida (55). Furthermore, pharmacological research has demonstrated that active constituents in dark plum (Wu Mei) modulate lipid metabolism via the PPAR and PI3K/AKT signaling pathways (56). Animal experiments by Wang Shaoping et al. have also confirmed that total Chaihu saponins from radix bupleuri (Chai Hu) can lower serum levels of TG, TC, and LDL-C, decrease malondialdehyde (MDA), and increase superoxide dismutase (SOD) activity, thereby elucidating its lipid-regulating mechanism through the modulation of genes such as ACC and HMGCR (57). Collectively, these findings provide a robust pharmacological foundation for our clinical observations.

The main advantage of this study is the combination of novel indicators, which for the first time reveals the multidimensional benefits of the Qingre Lishi decoction in obese patients with newly diagnosed T2DM. Despite the promising findings, this study has several limitations. Due to objective factors, the sample size was relatively small, so we expect to continue the series of multi-centre studies in the future. Furthermore, we plan to conduct long-term follow-up assessments to evaluate the sustained efficacy of the treatment and to refine the therapeutic strategy.

## 5 Conclusion

This study further elucidates that Qingre Lishi decoction not only provides effective glycemic control but also demonstrates multi-target therapeutic efficacy by optimizing body fat distribution, improving the lipid profile, and reducing novel metabolic risk indices (TyG, TyG-WC, TyG-BMI, and TyG-WHtR) in patients. Consequently, this work provides evidence-based medicine support for the capacity of the Qingre Lishi decoction to exert multi-target, multi-faceted, and systemic regulation in obese patients with newly diagnosed T2DM.

## Data Availability

The datasets presented in this article are not readily available because The original contributions presented in the study are included in the article/**Supplementary Material**. Further inquiries can be directed to the corresponding authors. Requests to access the datasets should be directed to gaotianshu67@163.com.
