# Supplementary material for: Effect of Qingre Lishi decoction on anthropometric and metabolic risk indices in obese patients with newly diagnosed T2DM: a real-world observational study
Source: Front Endocrinol (Lausanne). 2026 Jan 21;17:1721267. doi: 10.3389/fendo.2026.1721267 (PMC12867804; doi:10.3389/fendo.2026.1721267)
Supplement: Supplementary file 1 [file DataSheet1.docx]

**1 Supplementary Figures and Tables**

**1.1 Supplementary Figures**

**
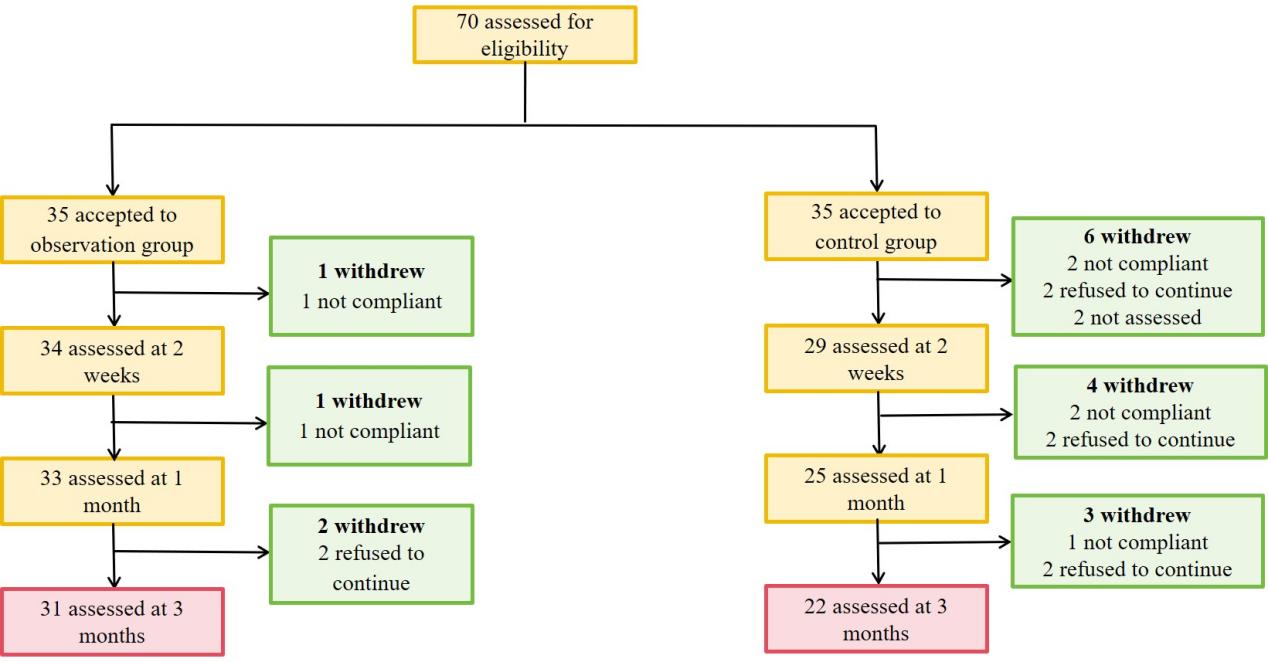
**

**Supplementary Figure 1** Progress of the research.

**1.2 Supplementary Tables**

**Table S1** Comparison of baseline characteristics between the observation and control groups [($\bar{\text{x}}$±s) or M (Ql, Qu)].

| **Parameters** | **Observation Group** | **Control group** | ***P* value** |
| --- | --- | --- | --- |
| Gender (*n*) |  | | |
| Male | 16 | 10 | 0.659 |
| Female | 15 | 12 |  |
| Age (years) | 45.77 ± 11.36 | 49.59 ± 12.09 | 0.246 |
| Course of disease (months) | 1.50 (0.50,12.00) | 1.00 (0.50,4.00) | 0.439 |
| BMI (kg/m^2^) | 27.00 ± 2.34 | 27.40 ± 2.49 | 0.549 |
| Education (0～12years/＞13years) |  |  |  |
| 0～12 | 12 | 13 | 0.143 |
| ＞13 | 19 | 9 |  |
| Physical activity intensity (*n*) |  |  |  |
| Low | 14 | 12 | 0.822 |
| Moderate | 12 | 7 |  |
| High | 4 | 3 |  |
| Smoking (*n*) |  |  |  |
| Yes | 7 | 2 | 0.197 |
| No | 24 | 20 |  |
| Drinking (*n*) |  | | |
| Yes | 9 | 6 | 0.889 |
| No | 22 | 14 |  |
| Family history of diabetes Genetic history (*n*) |  | | |
| Yes | 8 | 8 | 0.409 |
| No | 23 | 14 |  |

body mass index (BMI).

**Table S2** Comparison of basic biochemical indexes between observation and control groups [($\bar{\text{x}}$±s) or M (Ql, Qu)].

| **Parameters** | **Observation Group** | **Control group** | ***P* value** |
| --- | --- | --- | --- |
| C peptide  (ng/mL) | 3.20 (2.33,4.98) | 2.91 (1.97,3.29) | 0.159 |
| LDL-C  (mmol/L) | 2.83 ± 1.32 | 2.91 ± 0.99 | 0.805 |
| TG  (mmol/L) | 1.92 (1.25,3.25) | 1.94 (1.07,2.68) | 0.718 |
| ALT  (U/L) | 27.74 ± 13.21 | 29.86 ± 13.89 | 0.579 |
| AST  (U/L) | 25.06 ± 11.33 | 32.18 ± 11.92 | 0.034 |
| GGT  (U/L) | 24.84 ± 9.75 | 35.55 ± 11.99 | 0.001 |
| Scr  (μmol/L) | 61.71 ± 13.49 | 58.77 ± 12.85 | 0.426 |
| UREA  (mmol/L) | 5.60 (3.80,7.30) | 5.79 ± 1.28 | 0.752 |

low density lipoprotein (LDL-C), triglyceride (TG), alanine aminotransferase (ALT), aspartate aminotransferase (AST), γ-glutamyl transpeptidase (GGT), serum creatinine (Scr), urea nitrogen (UREA).

**Table S3** Comparison of the incidence of hypoglycemia between the observation group and the control group.

| **Parameters** | **Observation Group** | **Control group** | ***χ2*** | ***P* value** |
| --- | --- | --- | --- | --- |
| Hypoglycemia  [*n* (%)] | 2 (6.45) | 6 (27.27) | 4.353 | 0.037 |
